# Cross-cultural adaptation and validation of the Sinhala version of SarQoL^®^ for assessing quality of life in older women with sarcopenia

**DOI:** 10.1186/s12877-025-06137-8

**Published:** 2025-07-02

**Authors:** Nirmala Rathnayake, Thilina Abeygunasekara, Gayani Liyanage, Sewwandi Subasinghe, Warsha De Zoysa, Dhammika Palangasinghe, Sarath Lekamwasam

**Affiliations:** 1 Department of Nursing, Faculty of Allied Health Sciences, University of Ruhuna, Galle, Sri Lanka; 2 Department of Pharmacology, Faculty of Medicine, University of Ruhuna, Galle, Sri Lanka; 3 Department of Pharmacy, Faculty of Allied Health Sciences, University of Ruhuna, Galle, Sri Lanka; 4 Department of Medicine, Faculty of Medicine, University of Ruhuna, Galle, Sri Lanka

**Keywords:** Sarcopenia, Sinhala version, Quality of life, Questionnaire, Validation

## Abstract

**Introduction:**

Sarcopenia, characterized by the loss of muscle strength, mass, and function, significantly impacts the quality of life (QoL) in older adults. The Sarcopenia Quality of Life (SarQoL^®^) questionnaire is a disease-specific tool designed to evaluate QoL in individuals with sarcopenia. It has been cross-culturally adapted and validated in various languages worldwide. However, no validated tool existed in Sinhala, limiting the ability to assess QoL among Sri Lankan older adults with sarcopenia. This study aimed to cross-culturally adapt the SarQoL^®^ into Sinhala and evaluate its psychometric properties.

**Methods:**

The standard protocol for cross-cultural adaptation was followed, including forward translation, synthesis, backward translation, expert committee review, and pre-testing. The finalized Sinhala version of SarQoL^®^ was administered to a randomly selected sample of 295 older women (≥ 65 years) who regularly attended medical clinics at a tertiary care hospital in Sri Lanka. The validated Short Form 36 survey (SF-36) was used for concurrent validation. Probable sarcopenia was identified using handgrip strength (HGS) based on local cutoff values. SarQoL^®^ was re-administered via phone after two weeks to 34 women with probable sarcopenia to assess test-retest reliability. Psychometric properties, including internal consistency, reliability, construct validity, discriminant validity, and floor and ceiling effects, were evaluated.

**Results:**

The mean age of participants was 72.1 ± 4.8 years, with 139 (47.1%) identified as having probable sarcopenia. The questionnaire demonstrated strong internal consistency (Cronbach’s alpha = 0.82) and excellent test-retest reliability (Intraclass Correlation Coefficient; ICC = 0.90; 95% CI: 0.81–0.96). Significant correlations between SarQoL^®^ scores and SF-36 dimensions (r range: 0.24–0.75, *p* < 0.001) confirmed concurrent validity. Total SarQoL^®^ scores were significantly lower in sarcopenic women compared to non-sarcopenic women (54.24 ± 14.52 vs. 62.10 ± 15.31, *p* < 0.001), confirming discriminant validity. No floor or ceiling effects were observed.

**Conclusions:**

The Sinhala version of SarQoL^®^ is a reliable and valid tool for assessing QoL in older adults with sarcopenia. It can be effectively used in both clinical practice and research to evaluate QoL and guide interventions targeting sarcopenia in Sri Lanka.

**Trial registration:**

Not applicable.

## Background

Sarcopenia, characterized by the progressive loss of skeletal muscle mass, strength, and function, has emerged as a significant public health concern, particularly among aging populations [1]. The condition is associated with increased risks of adverse outcomes including physical disability, reduced quality of life (QoL), and higher mortality rate [2]. As the global population ages, the prevalence of sarcopenia is expected to rise necessitating effective interventions and tailored healthcare strategies [2].

Although sarcopenia affects both men and women, older women tend to experience greater disability due to several factors such as poor nutrition, low physical activity and the additional impact of menopause [3]. Assessing the impact of sarcopenia on health-related quality of life (HRQoL) is a critical aspect of patient care as it provides valuable insights into the condition’s effect on daily life and overall well-being, facilitating personalized treatment approaches [1].

Among the tools available for assessing sarcopenia-specific HRQoL, the Sarcopenia Quality of Life (SarQoL^®^) questionnaire has gained a wide recognition for its validity and reliability [4]. Designed specifically for individuals with sarcopenia, the SarQoL^®^ captures the physical, emotional, and social dimensions of the condition offering a comprehensive assessment [4]. The questionnaire consists of 55 items, arranged into 22 questions under major seven domains of dysfunction namely physical and mental health, locomotion, body composition, functionality, activities of daily living, leisure activities and fears [4]. The questionnaire has been translated into several languages including French, English and Romanian with each version demonstrating strong validity, reliability, and the ability to discriminate between sarcopenic and non-sarcopenic individuals in terms of HRQoL [5–7].

Despite its broad recognition, the SarQoL^®^ questionnaire is predominantly available in Western languages, which limits its applicability in non-English-speaking regions. It has also been translated into several Asian languages, including Thai, Hindi, Kannada, Bangla, Chinese, Malay, and Marathi (https://sarqol.org/en). Notably, the Kannada version has demonstrated high internal consistency and validity within its local context [8].

Sri Lanka is experiencing a rapidly aging population, with projections indicating that nearly one in four citizens will be aged 60 years or older by 2041 [9]. This demographic shift brings increased vulnerability to age-related conditions such as sarcopenia a progressive loss of skeletal muscle mass and function which significantly impairs mobility, independence, and QoL in older adults. Despite the growing public health importance of sarcopenia, its assessment in clinical and community settings remains limited in Sri Lanka. This is compounded by a lack of validated tools adapted for the local population. While English-language and other language assessment tools for sarcopenia exist, reliance on these can introduce inequities, especially in a multilingual society where a significant portion of the population speaks Sinhala or Tamil. Sinhala is the most widely spoken language in Sri Lanka, primarily among individuals from rural and lower socioeconomic backgrounds. If sarcopenia screening is conducted in English, speakers of other languages may face challenges in understanding and completing assessments, potentially resulting in misdiagnosis or exclusion from necessary interventions. Therefore, there is a critical need to culturally adapt and validate Sinhala version of the SarQoL^®^ that is essential to ensure holistic and contextually relevant care for individuals with sarcopenia. Hence, this study aimed to translate, culturally adapt, and validate the SarQoL^®^ questionnaire into Sinhala, adhering to the established guidelines for cross-cultural adaptation and psychometric validation.

## Methods

This validation study consisted of two phases including the cross-cultural adaptation and psychometric validation. Ethical approval for the study was obtained from the Ethics Review Committee of the Faculty of Medicine, University of Ruhuna, Sri Lanka (Ref no: 2021.P.016: 17.02.2021).

### Phase 1 – cross-cultural adaptation

The cross-cultural adaptation of SarQoL^®^ followed standard protocol guidelines to ensure linguistic and conceptual equivalence to the original instrument in English language [10]. This process outlined by the working committee of the SarQoL^®^ involved the following steps.

1. Forward translation: The initial translation of the original SarQoL^®^ questionnaire into Sinhala was carried out by two independent translators who were fluent in both English and Sinhala. To ensure both clinical relevance and linguistic accuracy, one translator was a healthcare professional familiar with sarcopenia-related terminology, while the other was a language expert with no medical background. This combination allowed for a balanced translation that preserved both the medical integrity and the cultural-linguistic nuances of the tool. The translators were explicitly instructed to avoid literal, word-for-word translation. Instead, they were guided to focus on conveying the intended meaning of each item in a culturally appropriate and contextually accurate manner. Each translator worked independently and completed the translation within the given time (two weeks). Along with their translations, they submitted detailed written reports highlighting any difficulties encountered such as ambiguous terms or culturally sensitive expressions and explained the reasoning behind their specific word choices. This process ensured that the translated version maintained conceptual equivalence with the original questionnaire.
2. Synthesis: A synthesized version of the two forward translations was developed through a collaborative discussion involving both translators and research team. This reconciliation meeting was conducted in the Department of Medicine, Faculty of Medicine, University of Ruhuna which takes about two hours. The session was attended by the principal investigator (NR), supervisor of the project (SL) and both translators. During the meeting, each question and item of the questionnaire were carefully reviewed to identify and resolve any discrepancies between the two translations. Particular attention was paid to variations that could indicate ambiguities or culturally inappropriate wording. The author actively documented all identified inconsistencies, especially those reflecting potentially unclear phrasing. Through detailed discussion, the team reached a consensus on each item, ultimately producing the first synthesized version of the Sinhala translation.
3. Backward translation: The synthesized Sinhala version of the SarQoL^®^ questionnaire was back-translated into English by two independent bilingual translators who were blinded to the original questionnaire. To ensure an unbiased interpretation, neither translator had a clinical background. According to the guidelines provided by the original developers, the preferred profile for back-translators includes having English as their first language. However, as it was not feasible to recruit such translators for Sri Lankan languages, an alternative approach was adopted in this study. Two experienced English teachers were engaged to perform the back-translation. These individuals had no medical background, further reducing the risk of bias. The translators independently completed the back-translations over a period of one month. The primary objective of this step was to verify that the Sinhala translation retained the conceptual equivalence and content of the original SarQoL^®^ questionnaire.
4. Expert committee review: An expert committee consisting of clinicians, linguists, and methodology experts and all the forward and backward translators reviewed all translations and synthesized their findings to ensure that the Sinhala version of the SarQoL^®^ questionnaire was both culturally and conceptually equivalent to the original instrument. Adjustments were made to enhance clarity and ensure relevance to the local context. Prior to the expert committee review, the author identified discrepancies between the back-translated version and the original questionnaire, and prepared a document detailing these discrepancies for discussion. The expert committee meeting was conducted at the Department of Medicine, Faculty of Medicine, University of Ruhuna. The methodology expert led the discussion, addressing and clarifying the identified discrepancies. The meeting lasted for two hours, during which the pre-test version of the Sinhala questionnaire was finalized.
5. Test of the pre-final version: The pre-final version of the Sinhala SarQoL^®^ was administered to a group of 30 older women (≥ 65 years) who presented with patients (as caregivers) at the medical clinics of National Hospital, Galle, Sri Lanka who identified as having pre-sarcopenia identified by low hand grip strength (HGS < 9.66 kg) [11]. This pilot study was done approaching individual women to evaluate comprehensibility and cultural relevance. All participants completed the pre-final version of the Sinhala SarQoL^®^ questionnaire in the presence of the principal investigator, with the average completion time being approximately 10–12 min. Following administration, face-to-face interviews were conducted with several participants selected randomly to gather feedback on any difficulties encountered such as complex words or phrases and to assess the cultural relevance and clarity of the questionnaire. Only minor adjustments were necessary to do, they were informed to the expert committee and relevant modifications were done accordingly.

### Phase 2 – psychometric validation

#### Study design, setting and sampling

The study sample of this phase consisted of older women aged 65 years or more who regularly attended medical clinics at the National Hospital, Galle which is the main tertiary care hospital in Southern Sri Lanka. Participants were recruited using systematic random sampling method. Inclusion criteria were: (i) female sex, (ii) age ≥ 65 years, (iii) regular attendance at the medical clinics and (iv) willingness to provide informed consent. Participants with severe cognitive impairment or any condition that precluded reliable communication were excluded.

Those who fulfilled the inclusion criteria were recruited for the study considering the sample size calculated considering the number of items in the questionnaire (1 item:5 participants ratio), and adding 10% to compensate for incomplete data [12]. Then the total sample size required for the study was 302 participants.

#### Final administration of the SarQoL^®^ questionnaire

The finalized Sinhala version of SarQoL^®^ was administered to 302 older women selected via systematic random sampling. A trained research assistant facilitated face-to-face interviews to ensure consistent administration and to assist participants who had difficulty in reading the questionnaire.

Seven datasheets had to be removed due to incomplete responses and the final analysis included data only from 295 women.

#### Concurrent administration of SF-36

To assess the construct validity, the Sinhala version of the SarQoL^®^ was administered alongside the validated Sinhala version of the Short Form 36 survey (SF-36), a widely used generic health-related QoL instrument. It measures eight health domains: physical functioning, role limitation due to physical problems, bodily pain, general health perception, vitality, social functioning, role limitation due to emotional problems, and mental health. In this questionnaire, each domain is assigned a score ranging from 0 to 100 using the original coding algorithm [13].

#### Assessment of sarcopenia

Probable sarcopenia was identified in accordance with the European Working Group for Sarcopenia of Older Adults II (EWGSOP II) criteria published in 2019 [1]. HGS (in kg) was measured using a calibrated digital dynamometer and the local cutoff values (< 9.66 kg) published earlier were used to determine probable sarcopenia [11]. Measurements were taken in the dominant hand while participants were seated and they were encouraged to exert maximal effort and the average of two consecutive measurements was taken for the analysis.

#### Re-administration of the SarQoL^®^ questionnaire

**Fig. 1:**
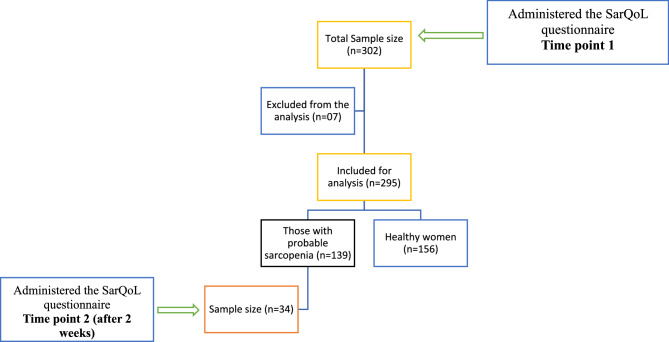
Selection of study participants for Phase 2

To evaluate the test-retest reliability of SarQoL^®^, the questionnaire was re-administered over the phone two weeks after the initial administration to a subset of randomly selected 34 women who were detected to have probable sarcopenia (low muscle strength). The interval of two weeks was chosen to minimize recall bias while avoiding significant changes in the participants’ health status [10] (Fig. 1).

#### Statistical analysis

Data were analyzed using SPSS version 25.0 software. Descriptive statistics were used to summarize demographic and clinical characteristics. The normality of data distribution was confirmed using the Kolmogorov-Smirnov test; therefore, parametric tests were employed for further analysis. A *p*-value of < 0.05 was considered statistically significant.

The validation of the Sinhala version of the SarQoL^®^ questionnaire was conducted in accordance with the measurement properties outlined in the COnsensus-based Standards for the Selection of Health Measurement Instruments (COSMIN) checklist [14], as well as the guidelines provided by the original tool developers. The validation process specifically assessed reliability, construct validity (including concurrent, convergent, and divergent validity), discriminative validity, and the presence of floor and ceiling effects. Psychometric properties evaluation was conducted to ensure the reliability and validity of the Sinhala version of the SarQoL^®^.

### Psychometric properties assessment

1. Internal Consistency reliability: This measures whether items within the questionnaire are consistent in measuring the same construct. Internal consistency was assessed using Cronbach’s alpha [15]. A Cronbach’s alpha value of 0.7 or higher was considered acceptable, indicating a satisfactory level of internal consistency [16].
2. Test-retest reliability: This evaluates the stability of the questionnaire over time by administering it to the same participants at two different points in time and measuring the agreement. Test-retest reliability was determined using the intraclass correlation coefficient (ICC) comparing the overall scores from two consecutive administrations of the SarQoL^®^ to ensure consistency over time [16]. An ICC over 0.70 was considered as acceptable [17].
3. Construct validity: Concurrent, Convergent and Divergent validities were evaluated to assess the concurrent validity.
  a Concurrent validity which evaluates how well a new measurement correlates with an established, already validated tool measuring the same construct was assessed by examining correlations between the total scores of SarQoL^®^ and SF-36 using the Pearson correlation [10].
  b The convergent validity [10] which assesses whether a measurement tool correlates strongly with other instruments that measure the same or similar constructs and the divergent validity [10] which assesses whether a measurement tool has low or negligible correlations with instruments measuring unrelated constructs were assessed by observing the correlations between SarQoL^®^ total score and domains of the SF-36. In these analyses, strong and positive correlations were expected between SarQoL and SF-36 domains reflecting physical and functional health. Pearson correlations were evaluated considering the distribution of data.
4. Discriminant validity: This was analysed by assessing the ability of SarQoL^®^ to differentiate between women with and without probable sarcopenia (low HGS; HGS < 9.66 kg) using independent samples t-tests.
5. Floor and ceiling effects: The presence of floor and ceiling effects was assessed by calculating the proportion of participants scoring the minimum and maximum possible scores, respectively, for the overall SarQoL^®^ score and its domains. Values > 15% were considered indicative of floor or ceiling effects [17].

### Consent and approvals

Relevant institutional approval obtained from the Director of the National Hospital, Galle. Informed written consent was obtained from all participants before data collection. Participants were assured of the confidentiality and voluntary nature of their participation, and they were free to withdraw at any point without consequences.

## Results

### Phase 1 - Cross-Cultural adaptation

The cross-cultural adaptation of the SarQoL^®^ questionnaire into Sinhala followed standard guidelines, ensuring linguistic and conceptual equivalence with the original version. The process included five steps: forward translation by two independent translators (one with a medical background), synthesis of translations, backward translation by two English language experts, expert committee review, and pilot testing. Discrepancies and culturally sensitive terms were discussed and resolved during synthesis and expert review meetings. The pre-final version was tested among 30 older women with probable sarcopenia at the National Hospital, Galle. All participants completed the questionnaire in 10–12 min. Feedback gathered through face-to-face interviews confirmed the clarity and cultural relevance of the tool, with only minor modifications made. The finalized Sinhala version was prepared based on this input.

### Phase 2 – psychometric validation

#### Characteristics of the study participants

A total of 295 older women participated in the study with mean age of 72.1 ± 4.8 years. Among them, 139 women (47.1%) were detected to have probable sarcopenia based on HGS measurements and the remaining 156 participants were classified as non-sarcopenic. Demographic characteristics and distribution of comorbidities, are summarized in Table 1.

**Table 1:** Basic characteristics of the study participants (*n* = 295)

Characteristic		Frequency (%)
Ethnicity	Sinhala	282 (95.6)
	Non-Sinhala	13 (4.4)
Civil status	Married	118 (40.0)
	Single	25 (8.5)
	Widowed/Divorced/Separated	152 (51.5)
Monthly income	Less than 20,000 LKR	261 (88.5)
	Between 20,000–50,000 LKR	29 (9.8)
	More than 50,000 LKR	5 (1.7)
Education level	Primary education	115 (39.0)
	Secondary education	149 (50.5)
	Tertiary education	31 (10.5)
Employment status	Unemployed	242 (82.0)
	Retired (not employed currently)	33 (11.2)
	Self-employed and employed	20 (6.8)
Characteristic		Mean (± SD)
BMI (Kg/m^2^)		21.92 (± 4.60)
Hand Grip Strength (Kg)		10.56 (± 4.75)
Comorbidities		Frequency (%)
Ischemic Heart diseases		87 (29.5%)
Diabetes Mellitus		92 (31.2%)
Dyslipidemia		224 (75.9%)
Stroke		3 (1.0%)
Respirators diseases (COPD, Asthma)		85 (28.8%)
Bone related disease (Osteoporosis, Osteoarthritis etc.)		110 (37.3%)
Thyroid related diseases		62 (21.0%)
Chronic Kidney Diseases		14 (4.7%)
BMI-Body Mass Index 300 Sri Lankan Rupees (LKR) ≈ 1 USD		

#### Reliability

The internal consistency of the Sinhala version of SarQoL^®^ was high (Cronbach’s alpha = 0.82), indicating the strong reliability of the questionnaire. Test-retest reliability, assessed in a subset of 34 women with probable sarcopenia, showed high degree of reproducibility with an ICC of 0.90 (95% CI: 0.81–0.96). These findings highlight the robustness of the tool for repeated use in the target population.

All individual dimensions of SarQoL^®^ showed significant correlations with the total SarQoL^®^ score, with correlation coefficients ranging from 0.39 to 0.91 (*p* < 0.001). These findings further support the internal validity of the questionnaire, demonstrating that the individual dimensions contribute meaningfully to the overall assessment of QoL (Table 2).

**Table 2:** Correlation of SarQoL^®^ domains scores with total SarQoL^®^ score (*n* = 295)

Domains of SarQoL^®^	Pearson correlation with Total Score of SarQoL^®^
Physical and mental health	0.83
Locomotion	0.86
Body composition	0.67
Functionality	0.91
Activities of daily living	0.85
Leisure activities	0.39
Fears	0.49

#### Construct validity

- *Concurrent validity* - The total SarQoL^®^ score demonstrated significant positive correlations with all dimensions of the SF-36 survey (r range: 0.24 to 0.75, *p* < 0.001) (Table 3).
- *Convergent and Divergent validity* - Stronger correlations were observed with the similar dimensions of SF-36 questionnaire related to physical functioning, role limitation due to physical health, energy pain and general health, indicating that SarQoL^®^ adequately reflects HRQoL, particularly in areas relevant to sarcopenia. Other items such as social functioning, role limitation due to the emotional health and emotional wellbeing showed weaker correlations with SarQoL^®^ indicating the divergent validity (Table 3).

**Table 3:** Correlation of SarQoL score and domains’ score of SF-36 (*n* = 295)

Validity	Domains of SF 36	Pearson correlation with Total Score of SarQoL^®^
Convergent validity (similar dimensions)	Physical functioning	0.75
	Role limitation due to physical health	0.56
	Energy/Fatigue	0.62
	Pain	0.67
	General health	0.54
Divergent validity (different dimensions)	Social functioning	0.34
	Role limitation due to emotional health	0.35
	Emotional wellbeing	0.24

- *Discriminant Validity* - The discriminant validity of SarQoL^®^ was confirmed by its ability to distinguish between women with or without probable sarcopenia. The mean total SarQoL^®^ score was significantly lower in women with probable sarcopenia (54.2 ± 14.5) compared to those without sarcopenia (62.1 ± 15.3) (*p* < 0.001) (Table 4).

**Table 4:** Comparison of SarQoL^®^ scores between older women with probable sarcopenia and without probable sarcopenia (*n* = 295)

Domains of SarQoL^®^	Sarcopenic women Mean (SD) (*n* = 139)	Non-sarcopenic women Mean (SD) (*n* = 156)
Physical and mental health	45.89 (15.51)	55.03 (15.51)
Locomotion	49.29 (22.27)	60.34 (25.27)
Body composition	52.11 (25.11)	57.23 (22.96)
Functionality	66.73 (15.72)	73.31 (14.61)
Activities of daily living	53.18 (18.58)	60.61 (17.30)
Leisure activities	20.10 (7.28)	26.44 (7.11)
Fears	72.81 (18.21)	78.31 (17.66)
Total score	54.24 (14.52)	62.10 (15.31)

#### Floor and ceiling effects

No floor or ceiling effects were observed for the total SarQoL^®^ score or its individual dimensions, as fewer than 15% of participants achieved the lowest or highest possible scores. No sarcopenic subject obtained the highest or lowest score for the questionnaire, both for the first (*n* = 139) and second SarQoL^®^ administration (*n* = 34).

## Discussion

This study aimed to culturally adapt and validate the SarQoL^®^ questionnaire into Sinhala and assess its psychometric properties among older women in Sri Lanka. The results demonstrate that the Sinhala version of SarQoL^®^ is a reliable and valid tool for measuring the QoL in older women with sarcopenia.

### Cultural adaptation

The cultural adaptation of the SarQoL^®^ into Sinhala highlights the importance of ensuring that HRQoL tools are linguistically and contextually appropriate for the target population. Sarcopenia, as a condition that affects physical, emotional, and social domains, requires a rigorous approach to capture the lived experiences of older adults in different cultural settings. In this study, cultural adaptation went beyond literal translation, incorporating local linguistic terms and cultural considerations to ensure the tool’s relevance and acceptability among Sinhala-speaking older women in Sri Lanka.

One aspect of adaptation involved addressing the nature of Sri Lankan society in which the family and community play a central role in the lives of older adults, influencing how they perceive and report their QoL. For an instance, questions related to emotional well-being and social interactions had to be modified to accommodate the interconnectedness of individuals with their families and communities. This was particularly important given the tendency of individuals in theses cultures to underreport personal challenges due to stigma [18].

Furthermore, the socioeconomic and healthcare disparities in Sri Lanka posed unique challenges during adaptation. Older adults in rural or resource-limited areas might face significant barriers to accessing healthcare and maintaining physical activity, which directly impacts their perception of HRQoL [19]. Adapting the SarQoL^®^ to include culturally relevant examples and scenarios helped bridging these gaps, ensuring that the tool would accurately captured the experiences of individuals in diverse settings, including urban and rural populations.

The adaptation process also considered linguistic simplicity and accessibility, as the target population included individuals with varying levels of literacy [20]. This was crucial for ensuring that older adults regardless of their educational background could understand and respond to the questionnaire. Hence, technical terms related to health and physical functioning were simplified or replaced with culturally familiar expressions.

### Reliability

The high internal consistency and test-retest reliability of the Sinhala SarQoL^®^ align closely with results from validation studies conducted in diverse cultural contexts, such as French [21], Polish [22], Spanish [23], Romanian [24], Greek [25], and Hangarian [26] populations. Similarities in reliability metrics across different studies can be attributed to the universal nature of the SarQoL^®^ tool’s design, which incorporates domains that are broadly relevant to sarcopenia’s impact on HRQoL.

The discrepancies observed in Cronbach’s alpha values across studies including slightly higher values in European populations [6] and in the Asian Kannada version [8] may reflect underlying cultural and contextual differences in how QoL items are perceived and interpreted. In high-income countries, individuals may have greater exposure to self-administered health surveys and better overall health literacy, contributing to more consistent and reliable responses. Conversely, in countries like Sri Lanka, disparities in health literacy, educational background, and socioeconomic status may influence participants’ understanding and interpretation of questionnaire items, thereby affecting internal consistency [27]. Furthermore, the inclusion of only women in our study may also have contributed to the variation in reliability scores, as gender-specific experiences and perceptions of sarcopenia-related QoL could differ.

The significant correlations between individual SarQoL^®^ dimensions and the total score demonstrate the comprehensiveness of the tool. Similar findings have been observed in studies conducted in Turkey [28] and Dutch [29] populations. These correlations suggest that the various domains of SarQoL^®^ are interrelated and collectively contribute to the holistic understanding of the QoL impairment caused by sarcopenia.

Possible causes for discrepancies in correlation strength between studies may include differences in cultural perceptions of health and QoL [30]. In Sri Lanka, older adults might prioritize physical independence and family support over emotional or social well-being, influencing their responses to certain questionnaire items [31].

### Concurrent, convergent and divergent validity

The significant correlations between the SarQoL^®^ total score and SF-36 dimensions reflect the tool’s ability to capture HRQoL dimensions, particularly those related to physical and functional health. These findings are consistent with previous studies, such as those conducted in Spanish [23] and French [21] populations, where the SarQoL^®^ showed strong associations with physical and functional health-related domains.

The strongest correlations in physical and functional health-related domains highlight the universal impact of sarcopenia on mobility, independence, and physical well-being [2]. However, discrepancies in correlation strength with social and emotional domains may reflect cultural differences. In Sri Lanka, family support structures and cultural norms around aging may lessen the perceived social and emotional impacts of sarcopenia compared to individualistic societies [31].

### Discriminant validity

The significantly lower SarQoL^®^ scores among sarcopenic women compared to non-sarcopenic women confirm the tool’s ability to distinguish between these groups. Similar findings have been reported in other studies [6], reinforcing SarQoL^®^’s ability in identifying QoL impairments associated with sarcopenia.

The impact of sarcopenia on a wide range of domains such as physical, emotional, and social dimensions highlight its multifaceted nature. Differences in mean scores across studies may be explained by variations in lifestyle, nutrition, access to healthcare, and cultural perceptions of aging [32]. The dietary diversity and prevalence of malnutrition in Sri Lanka could contribute to more severe QoL impairments in sarcopenic individuals compared to higher-income countries [33].

### Absence of floor and ceiling effects

The lack of floor and ceiling effects in the Sinhala SarQoL^®^ ensures its sensitivity to detect variations in quality of life across different severity levels of sarcopenia. This finding is consistent with validation studies conducted in Europe populations [6] and Kannada population [8], where the absence of such effects was highlighted as a strength.

### Implications

The validation of the Sinhala SarQoL^®^ is a significant milestone in addressing the burden of sarcopenia in Sri Lanka, particularly with its rapid aging population and limited healthcare resources. Its cultural adaptability ensures relevance to local contexts, making it a valuable tool for healthcare providers, policymakers, and researchers. SarQoL^®^ can guide clinical practices by monitoring disease progression, evaluating interventions, and creating holistic care plans. It supports community health initiatives through targeted screenings, education, and awareness programs, especially in rural and underserved areas. Additionally, the tool can inform national policies promoting healthy aging, such as nutrition and physical activity programs, while facilitating international comparisons and cross-cultural research. By addressing physical, emotional, and social dimensions of sarcopenia, SarQoL^®^ helps design personalized interventions and identify vulnerable populations, ensuring equitable and effective management of aging-related health challenges in Sri Lanka.

### Strengths, limitations and recommendations

The validation of the Sinhala SarQoL^®^ questionnaire demonstrates significant strengths, including its rigorous cultural and linguistic adaptation to suit Sri Lanka’s context and its comprehensive evaluation of psychometric properties, such as reliability, validity, and discriminatory power. The study provides a standardized, culturally relevant measure for sarcopenia research and clinical practice, enabling healthcare professionals to design targeted interventions for the aging population. By addressing a critical gap in sarcopenia assessment, the Sinhala SarQoL^®^ lays a solid foundation for improving patient care, supporting policy development, and promoting healthy aging in Sri Lanka.

However, the study has several limitations to acknowledge. The validation was conducted only among older women with probable sarcopenia due to the unavailability of locally validated cutoff values for males. As a result, the findings cannot be generalized to the male population at this stage. Secondly, the assessment was limited to individuals with probable sarcopenia, as advanced diagnostic techniques for confirming sarcopenia (such as Dual Energy X Ray Absorptiometry or Bio Impedance Analysis) are not readily accessible in Sri Lanka due to resource constraints. The use of probable sarcopenia, as permitted by the EWGSOP guidelines, was a pragmatic decision in the context of these limitations.

Its cross-sectional design restricts assessment of responsiveness to interventions over time, and reliance on self-reported data introduces potential recall and response biases. The weaker correlations with emotional and social domains may reflect cultural barriers, and resource constraints in LMICs, along with the absence of comparisons with objective measures of sarcopenia like muscle mass, further limit its practical applicability.

Further, according to the validation protocol of the SarQoL^®^ questionnaire, the assessment of construct validity requires the use of two QoL measurement tools. However, in our study, we utilized only a single instrument, the SF-36 survey for this purpose. We acknowledge this as a limitation, and recommend that future evaluations incorporate an additional QoL tool to further strengthen the assessment of construct validity. Future studies should address these issues by expanding sample diversity, conducting longitudinal studies, integrating objective measures, and validating the tool in other cultural and linguistic groups, such as Tamil speakers. Raising awareness among policymakers and training healthcare providers on the burden of sarcopenia will be critical for effectively integrating the SarQoL^®^ into national health strategies in resource-limited settings. Despite its limitations, the Sinhala SarQoL^®^ is a reliable and culturally relevant tool with the potential to enhance sarcopenia management in Sri Lanka.

## Conclusions

The cultural adaptation of the Sinhala SarQoL^®^ emphasizes the critical need to adapt HRQoL instruments to align with the linguistic, social, and cultural contexts of the target population. This adaptation ensures accurate measurement of sarcopenia’s impact on quality of life, enhancing its applicability in clinical and research settings in Sri Lanka. The Sinhala SarQoL^®^ has proven to be a reliable, valid, and culturally appropriate tool, particularly for assessing the quality of life among older women. Its strong psychometric properties meet international standards, supporting its use in both practice and research. Expanding similar efforts to other cultural and linguistic groups, such as Tamil speakers, will further extend its utility and inclusivity. By providing a standardized measure of quality of life, the Sinhala SarQoL^®^ holds significant potential to deepen the understanding of sarcopenia’s impact and contribute to improved patient care and health strategies in Sri Lanka.

## Data Availability

The data used to support the findings of this study are available from the corresponding author upon request.

## Abbreviations

QoL: Quality of life
HRQoL: Health related Quality of Life
SarQoL®: Sarcopenia Quality of Life
SF: 36-Short Form 36 survey
HGS: Handgrip strength
ICC: Intraclass correlation coefficient
LMIC: Low-and Middle-income countries
EWGSOP II: European Working Group for Sarcopenia of Older Adults II
